# Atypical working hours are associated with substance use, especially in women: longitudinal analyses from the CONSTANCES cohort

**DOI:** 10.1192/j.eurpsy.2022.242

**Published:** 2022-09-01

**Authors:** N. Hamieh, G. Airagnes, A. Descatha, M. Goldberg, F. Limosin, Y. Roquelaure, C. Lemogne, M. Zins, J. Matta

**Affiliations:** 1 INSERM UMS 011, Population-based Epidemiological Cohorts Unit, Villejuif, France; 2 Université de Paris, School of Medicine, Faculty Of Health, Paris, France; 3 AP-HP.Centre-Université de Paris, Dmu Psychiatrie Et Addictologie, Paris, France; 4 Academic Hospital CHU Angers, Poison Control Center, Angers, France; 5 Université de Paris, AP-HP, Hôpital Corentin-Celton, Dmu Psychiatrie Et Addictologie, ISSY LES MOULINEAUX, France; 6 University of Angers, Centre Hospitalier Universitaire d’Angers, Université de Rennes, Centre De Consultations De Pathologie Professionnelle Et Santé Au Travail, Angers, France; 7 AP-HP, Assistance Publique - Hôpitaux de Paris, Adult Psychiatry, Paris, France

**Keywords:** Atypical working hours, substance use, Epidemiology, Sugar and fat

## Abstract

**Introduction:**

Difficult working conditions could be associated with addictive behaviors.

**Objectives:**

To examine the prospective associations between atypical working hours and substance use, including sugar and fat consumption.

**Methods:**

In the CONSTANCES cohort, a total of 47,288 men and 53,324 women currently employed were included from 2012-2017 for tobacco and cannabis outcomes, and 35,647 and 39,767, respectively from 2012-2016 for alcohol and sugar and fat outcomes, and they were then followed up annually. Atypical working hours were self-reported at baseline and considered three different indicators: night shifts, weekend work and non-fixed working hours. Generalized linear models computed odds of substance use and sugar and fat consumption at follow-up according to baseline atypical working hours while adjusting for sociodemographic factors, baseline depression and baseline level of consumption.

**Results:**

Night shifts increased significantly the odds of using tobacco in women (Odds ratios, ORs varying from 1.55 to 1.62) and cannabis in men (ORs varying from 1.80 to 1.95). Weekend work increased the odds of using tobacco (ORs varying from 1.51 to 1.67) and alcohol (OR of 1.16) in women. Non-fixed working hours increased the odds of using tobacco and alcohol in men and women (ORs varying from 1.15 to 1.19 and 1.12 to 1.14, respectively). Dose-dependent relationships were found for tobacco use in women (P for trends<0.0001). No significant associations were found for sugar and fat consumption.

**Conclusions:**

The role of atypical working hours on substance use should be taken into account by public health policy makers and clinicians for information and prevention strategies, especially among women.

**Disclosure:**

Nadine Hamieh was supported by a grant from “Direction de la recherche, des études, de l’évaluation et des statistiques”, DREES, Ministry of Labour, France.

